# Influence of Gestational Age on the Level of Functional Peptides (Peptidome) in Breast Milk

**DOI:** 10.3390/nu17172724

**Published:** 2025-08-22

**Authors:** Anna-Lena Abels, Johanna Ruhnau, Till Ittermann, Manuela Gesell Salazar, Anja Lange, Antje Vogelgesang, Hans Jörgen Grabe, Uwe Völker, Matthias Heckmann, Elke Hammer

**Affiliations:** 1 Department of Neonatology and Paediatric Intensive Care, University Medicine Greifswald, 17475 Greifswald, Germany; anlena.5732@gmail.com (A.-L.A.); matthias.heckmann@med.uni-greifswald.de (M.H.); 2 Department of Neurology, University Medicine Greifswald, 17475 Greifswald, Germany; antje.vogelgesang@uni-greifswald.de; 3 Institute for Community Medicine, University Medicine Greifswald, 17475 Greifswald, Germany; till.ittermann@uni-greifswald.de; 4 Department of Functional Genomics, Interfaculty Institute for Genetics and Functional Genomics, University Medicine Greifswald, 17475 Greifswald, Germany; manuela.gesell@uni-greifswald.de (M.G.S.); voelker@uni-greifswald.de (U.V.); hammer@uni-greifswald.de (E.H.); 5 Department of Psychiatry and Psychotherapy, University Medicine Greifswald, 17475 Greifswald, Germany; hans.grabe@med.uni-greifswald.de; 6 German Centre for Child and Adolescent Health (DZKJ), Partner Site Greifswald/Rostock, 17475 Greifswald, Germany

**Keywords:** human breast milk, peptidome, LC-MS/MS, preterm infant, term infant

## Abstract

Background/Objectives: Human milk provides essential nutrients and immune factors with beneficial impact on term, but especially preterm infants’ development. Therefore, this study focuses on the quantification of differences in the peptidome composition of breast milk from mothers of preterm and term infants, keeping in mind that this could reflect different biological needs of these infants or indicate nutritional gaps for healthy development. Methods: In a prospective observational study, breast milk samples were collected from 10 mothers of preterm infants (29–36 weeks gestational age,) and 13 mothers of term infants (37–41 weeks) at day 4 to 6 postnatally. A non-targeted tandem mass spectrometry approach was employed to analyze the milk peptidome. Results: In total, 4570 peptides were quantified. Adjusting the results for maternal age, weight, and height revealed a significant difference for 130 peptides derived from 19 different proteins between preterm and term milk. Proteins comprised high abundant proteins (e.g., α_S1_-casein, κ- casein, or ß-casein), but also proteins that are less prominent in milk but of high functional importance (e.g., Hypoxia-inducible factor 1-alpha, Olfactory receptor 4M1). The differentially abundant peptides included peptides derived from ß-casein, which have already been described as being involved in antimicrobial functions as well as proliferation stimulating. For another 32 peptides, bioactivity was predicted. Conclusions: The current study provides a comprehensive overview on the differences in the milk peptidome at different gestational ages independent from common maternal phenotypes and improved the database for future peptide functionality studies.

## 1. Introduction

Breast milk is the primary source of nutrition for preterm and term newborns. In addition to essential nutrients, milk also provides a range of functional components, including bioactive proteins. The degradation of milk proteins releases peptide fragments whose biological effects may differ from those of the original protein. [1]. Peptides in breast milk can be released from milk proteins by native proteases [2], but also through fermentation and during digestion. Whether these peptides have a bioactive effect depends on whether they reach their site of action. In the intestine, for example, opioid peptides can bind to intestinal opioid receptors and alter the motility of the gastrointestinal tract. Antimicrobial peptides can inhibit the growth of pathogens. Peptides that enter the bloodstream have systemic effects, such as ACE-inhibiting peptides, which lower blood pressure. For example, some milk peptides have been detected as fragments of κ- and α_S1_-casein in the blood of adults up to eight hours after a meal containing milk or yogurt [3]. Due to their development in breast milk, functional milk peptides could have several advantages for the nutrition of newborns compared to pharmaceuticals. They may exhibit superior safety and target selectivity compared to low-molecular-weight drugs, attributable to a reduced propensity for off-target interactions and consequent adverse effects. Peptides are particularly interesting as antimicrobial agents because their mechanisms of action are less likely to lead to the development of resistance [4].

Infants not being breastfed show a higher risk for suffering from infectious diseases such as gastroenteritis, otitis media or pneumonia as well as a higher risk for obesity, diabetes, and sudden infant death syndrome (SIDS) [5]. Especially preterm infants show an elevated risk for necrotizing enterocolitis (NEC) and late onset sepsis [6,7]. That marks the importance of understanding the bioactive components in breast milk. This knowledge would be essential for the production of artificial milk to help mothers who are unable to feed their children with breast milk. Mass spectrometry-based approaches enable the identification of these peptides from breast milk and a breast milk peptidome database is now also available, which facilitates the comparison and analysis of peptidome data [8].

Furthermore, functional peptides have also been studied in the breast milk of premature infants. Wan et al. (2013) [9] reported 419 peptides identified by MS/MS in colostrum. The identified peptides were found to originate from 34 proteins. Differences between the breast milk of mothers with term- and preterm-born infants were found for 41 peptides. A total of 23 peptides were present at higher levels in preterm milk, and 18 were present at higher levels in term milk. Dallas et al. (2015) [10] investigated human milk samples from term and preterm infants at four different stages of lactation. Preterm milk peptide counts, ion abundance, and concentration were significantly higher in preterm milk than term milk, while there was considerable overlap in the peptide patterns between the groups. An analysis using samples from the Dutch human milk bank identified a total of 16 different parent proteins from human milk, with no differences by gestational age or lactation state [11]. The absolute number of the 1104 endogenous peptides differed by gestational age and lactation state. However, there were no significant differences between the groups in those 32 peptides which had a predicted bioactive functionality. Maternal stress can also affect the concentration of certain peptides in breast milk, as shown by studies on endorphin concentrations in colostrum [12].

Human milk is a dynamic, bioactive fluid containing a complex array of nutrients, immune factors, and signaling molecules tailored to the developmental needs of the infant. Particularly for preterm infants, human milk is strongly recommended, as it has been shown to reduce the risk of severe complications such as necrotizing enterocolitis [13]. Given its critical role, this study focuses on the quantification and characterization of peptides to determine differences in the breast milk peptidome between mothers of preterm and full-term infants. These differences may reflect distinct biological requirements in early development or point to potential nutritional gaps in preterm milk that could impact health outcomes.

## 2. Materials and Methods

### 2.1. Samples

Breast milk samples from 13 and 10 mothers with term and preterm infants, respectively, were collected between 4 and 6 days after delivery. The composition of breast milk changes during the postnatal period, allowing a distinction to be made between colostrum in the first three days, the later transitional milk, which we focused on in our study, and mature breast milk two weeks after birth. Milk samples were collected from one expression in the neonatal or obstetric ward of the University Medicine of Greifswald between 8 a.m. and 10 a.m. Before sampling, the first droplets of milk were hand expressed, and afterwards the breast was cleaned using water and a washcloth, and samples of 20 mL were collected using clean electric breast pumps (Medela Medizintechnik, Dietersheim, Germany). The breast milk was collected into plastic tubes and transferred to the analytical center within an hour, divided into 2 mL aliquots and stored at −80 °C until analysis to yield optimal storage conditions [14]. The study was approved by the ethics committee of the University Medicine of Greifswald (reference number BB 050/19). The attending physician informed the parents about the study to obtain written parental consent. In the group of the mothers with term infants, the gestational age varied between 37 and 41 weeks and the birth weight between 2690 g and 4530. In the group of the mothers with preterm infants the gestational age was between 29 and 36 weeks and the birth weight ranged from 1480 to 2913 g. An overview of the characteristics of mothers and newborns is provided in Table 1.

### 2.2. Sample Preparation

Sample preparation was carried out as described by Howland et al. (2020) with modifications [14]. Briefly, breast milk samples were thawed for 10 min at RT followed by 20 min in an ice/water bath at 0 °C. Samples were centrifuged at 17,000× *g* at 4 °C for 30 min before the lipid layer accumulating on the top was removed mechanically with a spatula. The liquid phase was re-homogenized using a Vortex-blender, prior to the sampling of 100 µL aliquots. A total of 25 µL of methanol was added and samples vortexed prior to resting for 20 min at RT. Proteins were removed by filtration through a VivaCon500 filter (Sartorius, Göttingen, Germany, Ref. VN01H01, molecular weight cut-off 10 kDa, 14,000× *g*, RT, 30 min). The flow-through was frozen at −80 °C and lyophilized. Samples were reconstituted in 10 µL 1% acetic acid before small molecules were removed by the binding of peptides on C18 material (ZipTip 5 µg Tip C-18, Millipore Cooperation, Billerica, MA, USA) according to the manufacturer’s recommendation.

### 2.3. Mass Spectrometry and Database Search

Native peptides were analyzed by tandem mass spectrometry in data dependent mode using a nanoHPLC (Ultimate 3000, Thermo Electron, Bremen, Germany) coupled to an Exploris 480 mass spectrometer (Thermo Fisher Scientific, Waltham, MA, USA). MS data were analyzed via Sequest HT algorithm implemented in Proteome Discoverer 2.4 (Thermo Scientific, Bremen, Germany). An unspecific search was carried out against a reduced human Uniprot database. This database comprised protein sequences reported in Howland et al. (2020) and further expanded with proteins identified via unspecific peptide searches (Uniprot v 2022_04) conducted on the individual samples of all mothers (*n* = 2243) [14]. Carbamidomethylation at cysteine was set as static modification, and oxidation at methionine and conversion of *N*-terminal glutamate to pyroglutamate were defined as variable modification as well as acetylation at the protein *N*-terminus. Peptides with a minimum of 6 amino acids, FDR value 0.05, and identified in a minimum of 5 samples per phenotype group were considered for further analyses. A detailed description of data acquisition (Table S1A) and search parameters (Table S1B) are provided as Supplementary Materials.

### 2.4. Statistics

Characteristics of the mothers were reported as mean and standard deviation for continuous data or as absolute numbers and percentages for categorical data stratified by groups of gestational age. Peptides, which were available in less than five mothers in any of the groups were excluded from analysis. This resulted in 4570 peptide sequences available for analysis. Peptide intensities were log- and z-transformed before association with the group status (pre-term vs. term) in separate linear regression models adjusted for age, body height, and body weight. From these analyses, we derived a β-coefficient, a 95% confidence interval, and a −log-*p*-value for each peptide sequence. The peptide sequences were ordered according to their −log-*p*-value. After correction for multiple testing using the Bonferroni approach a −log-*p*-value > 4.96 was considered as statistically significant. The results were also visualized in a Volcano plot, in which the −log-*p*-values were plotted against the β-coefficients. In a similar manner we analyzed the data of 107 proteins. Here, proteins with −log-*p*-values > 3.33 reached statistical significance. All analyses were carried out with STATA 19.5 (Stata Corporation, College Station, TX, USA).

## 3. Results

According to the group classification, the gestational age and, consequently, the birth weight of the newborns differed. Although no difference in the Apgar score was observed after 1 min, the score was significantly lower in preterm infants after 10 min. Mothers of full-term infants tended to have a higher BMI (Table 1) but did not differ from mothers of preterm infants in other parameters, e.g., diabetes, complications during pregnancy, or in the number of c-sections. As there was an equal number of male and female newborns in the groups, any differences between the preterm and term group in the peptide composition should be independent of sex.

Following MS/MS analysis, a total of 12,011 unique peptide sequences were identified. It is already known from previous studies that the protein and peptide composition of breast milk is highly variable [15]. In line with these findings, filtering peptide sequences to those which could be identified in at least five breast milk samples in one of the sample groups reduced the number of unique peptide sequences to 4570. Assigning these peptide sequences to their respective proteins revealed that the majority were associated with the parent proteins β-casein (2.266), α_S1_-casein (415), and κ-casein (205).

**Figure 1:**
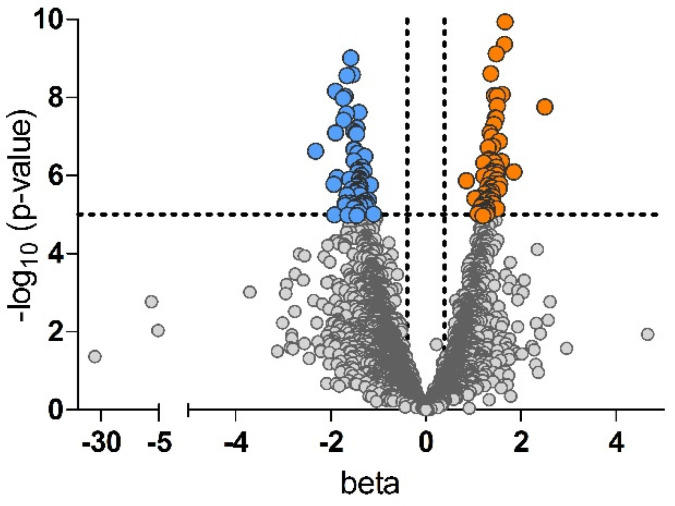
Volcano plot of the peptides in breast milk. Peptides with significantly (Bonferroni corrected *p*-value < 4.96 × 10^−4^) higher intensity in milk from mothers with preterm birth compared with term birth are shown in orange, peptides with lower intensities are shown in blue, gray color indicate, that peptides do not differ significantly between groups.

To highlight potential differences between preterm and term milk, the results were adjusted for possible covariates of the mother such as height, weight, and age. Following Bonferroni correction, 130 peptide sequences were identified as being significantly different (*p* < 4.96 × 10^−4^) between preterm and term breast milk samples (Supplemental Tables S2 and S3). While the significance of differences in the peptide composition varied, the effect size observed was rather small (Figure 1).

### 3.1. Peptide Sequences

**Figure 2:**
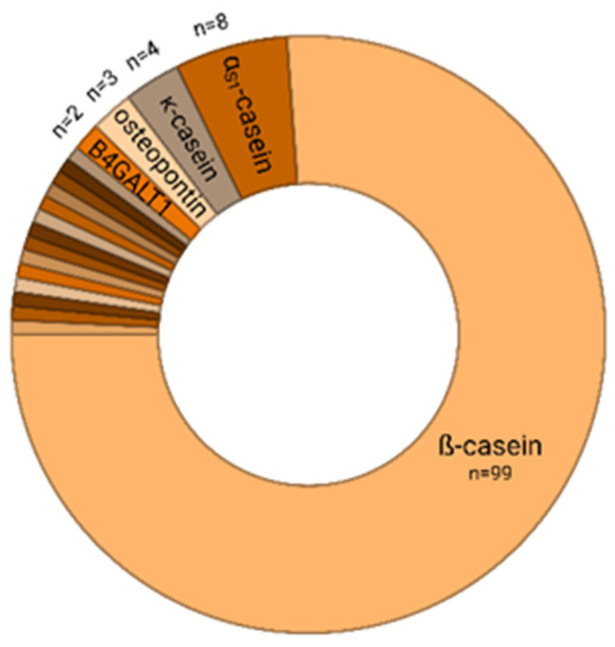
Contribution of allocated parental proteins to differential peptides with a significant Bonferroni corrected *p*-value < 4.96 × 10^−4^. Of 130 peptides with differential abundance in milk samples of mothers with term and preterm infants, 99 were derived from ß-casein. For four other proteins (labeled), more than one peptide displayed differential intensities, whereas 14 proteins contributed single peptides (not labeled, Supplemental Table S2). B4GALT1: β-1,4-galactosyltransferase 1.

Roughly the same number of peptides displayed higher intensities in milk of mothers of preterm (*n* = 68) and of term infants (62), respectively (Supplemental Table S2, Figure 1). In line with the total peptide composition, the majority of the 130 significantly different peptide sequences were allocated to the parent protein β-casein (*n* = 99). Further proteins represented with more than one sequence were α_S1_-casein (*n* = 8), κ-casein (*n* = 4), osteopontin (*n* = 3), and β-1,4-galactosyltransferase 1 (*n* = 2) (Figure 2).

**Figure 3:**
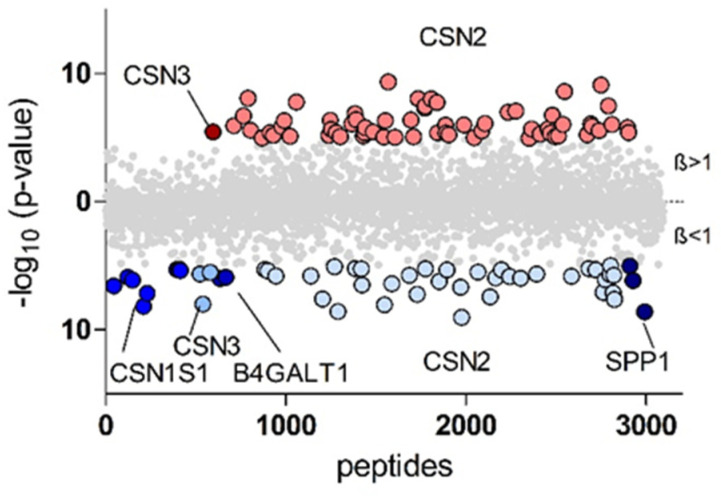
Peptides of proteins for which more than one peptide with significantly different intensities was observed. Shown are −log10 (*p* values) of peptides with higher (red) or lower (blue) intensities in breast milk from mothers with preterm infants. The gray color indicates no difference between sample groups. CSN1S1—α_S1_-casein, CSN3—κ- casein, B4GALT1—beta-1,4-galactosyltransferase 1, CSN2—ß-casein, SPP1—osteopontin.

Beside peptides assigned to ß-casein (*n* = 62) and κ-casein (*n* = 1) only four peptides were found to have a significantly higher intensity in preterm breast milk (positive beta value, Table 2). Interestingly, among those sequences, a peptide of the hypoxia-inducible factor 1-alpha (HIF-1a) was observed. This peptide at position 310–329 belongs to the PAS-associated *C*-terminal domain (PAC) of the protein and is involved in protein–protein interaction of the transcription factor HIF-1a. The biological importance of this finding has to be further investigated in future studies. Furthermore, peptides that exhibited higher intensity in term milk were derived from a greater number of distinct proteins (Figure 3, Supplemental Table S2).

Proteins were analyzed based on the sum of native non-tryptic peptides in the samples. In total, differences were observed for 11 proteins (Supplemental Table S3). Here, for some proteins, the results were driven by significant peptide differences. For example, two peptides were quantified for Lactoperoxidase, both with a positive beta indicating higher levels in preterm samples than in term sample, but one of the two displayed a significant Bonferroni corrected *p*-value (*p* < 4.96 × 10^−4^). In contrast, a significant result but with very low beta (1.05) was observed for ß-casein derived from peptides with positive as well as negative beta.

### 3.2. Functionality

Linking our findings with the Milk Bioactive Peptide Database [8] (https://mbpdb.nws.oregonstate.edu/; accessed on 13 August 2025) highlighted a potential biochemical functionality for two differentially abundant peptide sequences: QELLLNPTHQIYPVTQPLAPVHNPISV identified as being antimicrobial, and SPTIPFFDPQIPK which was found to stimulate proliferation. Both peptides originating from ß-casein were quantified in all samples of preterm and term breast milk, and were significantly higher in term breast milk.

Since potential biochemical functionalities of peptide sequences are still not fully understood, the potential for the bioactive functionality of the 130 peptide sequences identified was additionally assessed using the Peptide Ranker database [16] (http://distilldeep.ucd.ie/PeptideRanker/; accessed on 13 August 2025). This evaluation showed 35 peptide sequences ranking higher than 0.5 for bioactive functionality, as shown in Table 3.

## 4. Discussion

The ingestion of breast milk has been demonstrated to exert a beneficial influence on neonatal development, especially for preterm infants who must grow rapidly despite immature organ systems and a high risk of complications such as respiratory distress syndrome (RDS), NEC, and retinopathy of prematurity (ROP) [17,18]. Human milk supports this development not only through nutrients but also through bioactive compounds, including peptides that influence immune, metabolic, and developmental processes. Our study investigated whether gestational age affects the early breast milk peptidome, offering a reference point for future studies in more vulnerable preterm populations. Using a standardized, enzyme-free analytical protocol developed within the PriVileG-M-study, we identified 4570 unique peptides, with 130 significantly differing in abundance between term and preterm samples, with roughly equal numbers displaying higher intensities in preterm milk (68) and term milk (62), respectively. As in previous studies, most originated from β-casein, reinforcing its role as a major source of bioactive milk peptides. Our dataset is considerably larger and exhibits greater statistical robustness than those used in previous studies.

Wan et al. (2013) analyzed colostrum from preterm and term milk and detected 41 differential abundant peptides [9]. Among those, we found an overlap of 4 ß-casein derived peptides with two showing the same differences and two an opposite direction. The analysis of the pooled samples by Wan et al. and the known individual differences in the peptidome as well as expected differences between colostrum and breast milk at later timepoints might explain this result. Dallas et al. (2015) found five times more peptides in preterm than term milk, highlighting increased proteolytic activity [10]. The current study could not confirm this result. Although preterm milk contained a more consistent number of peptides (3620 ± 435) than term milk (3390 ± 932 peptides), no significant differences were found. Dingess et al. (2017) identified 1104 peptides, but after filtering for 50% presence across all samples for analysis in more detail, only 492 remained [11]. Changes were noted across both gestational age and lactation stage. All three studies consistently found β-casein to be the dominant precursor, but their sample sizes, gestational age groups, and timepoints varied widely. Our stricter sample selection, e.g., 4–6 days postpartum but also the use of a mass spectrometer with high sensitivity may explain the higher peptide count and differential resolution. In addition, Ombra et al. (2008) did not profile peptides but showed population differences in bioactive compounds (β-endorphin), supporting the idea that maternal factors and ethnicity may influence milk composition [12].

A particularly novel result in our study was the increased abundance of a HIF-1α-derived peptide in preterm milk. HIF-1α is crucial for fetal adaptation to hypoxia and regulates genes involved in angiogenesis (e.g., VEGF), erythropoiesis, and epithelial integrity [19,20,21]. Its loss after preterm birth—due to exposure to high oxygen—has been linked to bronchopulmonary (BPD), NEC, and ROP. The detection of HIF-1α fragments in milk suggests a possible exogenous route of extending fetal hypoxia-adaptive signaling. This peptide has not been reported in previous studies and could offer a novel protective mechanism warranting functional validation. In contrast, two β-casein-derived peptides—QELLLNPTHQIYPVTQPLAPVHNPISV and SPTIPFFDPQIPK—were significantly more abundant in term milk. These are known to have antimicrobial and proliferative effects. While detected in prior datasets, they were not described as differentially abundant between gestational age groups. If their functional roles are confirmed, their relative absence in preterm milk could justify peptide-based supplementation.

Due to the extensive variety of peptides present in breast milk, it is challenging to obtain empirical evidence for the biological functionality of all of them. This underscores the critical importance of the utilization of predictive tools for the purpose of predicting biological activity based on amino acid sequences. In this study, we used Peptide ranker to obtain more detailed information. This tool facilitates the preliminary selection of amino acid sequences from the 130 differentially abundant peptides, thereby enabling a more comprehensive investigation. Dingess et al. (2017) used PeptideRanker to gain information on the biological function of peptides, too, but in contrast to the results of the current study, they could not identify differences in peptides with annotated/predicted function related to gestational age [11]. More detailed information on target peptides may be obtained by working with synthetic peptide sequences. Alternatively, it is warranted to undertake an in-depth comparative analysis between differentially abundant peptides and homologous short sequences characterized by bioactive functionality. While these short sequences are present in a significant number in the Milk Bioactive Peptide Database, they often cannot be reliably identified and quantified by LC-MS/MS.

The differences between studies are likely driven by biological factors (e.g., gestational age definitions, maternal health), technical variability (sample preparation, peptide isolation), and timepoint heterogeneity. Our protocol minimized preanalytical digestion artifacts, and samples were collected during transitional milk to reduce variability. However, our study remains limited by a modest sample size (term *n* = 13, preterm *n* = 10) and a single early timepoint. Future research should include longitudinal designs to assess whether bioactive peptides abundant in term milk emerge later in preterm lactation, potentially compensating for early deficits. Finally, while peptide functions were predicted using established databases, experimental confirmation—especially for the HIF-1α peptide and the term-associated antimicrobial sequences—is essential to translate these findings into clinical application.

## 5. Conclusions

In conclusion, our study reinforces the concept of gestational-age-specific differences in the breast milk peptidome. From a well-selected sample set of milk from term and preterm mothers analyzed by a sensitive mass spectrometry-based method, the current study provides a comprehensive overview on the differences in the milk peptidome at different gestational ages independent from common maternal phenotypes. Due to the equal number of samples from mothers with male and female infants included in the study, our findings can be considered unrelated to the sex of the newborns. Our results indicate the importance of the use of predictive tools to identify peptides with biofunctionality, thereby expanding the known repertoire of potentially functional peptides, and highlight new candidates for nutritional support in preterm infants.

## Figures and Tables

**Table 1:** Description of mothers and their infants included in the study. The data are separated by the categorization of preterm and term birth of the infants.

	Term	Preterm	*p*-Value
*N*	13	10	
Age of the mother ^a^	33.54 [5.2]	30.40 [6.0]	0.26 ^c^
Maternal height in m ^a^	1.68 [0.08]	1.72 [0.05]	0.21 ^c^
Maternal weight in kg ^a^	78.08 [15.0]	67.76 [6.8]	0.15 ^c^
Body mass index in kg/m^2 a^	27.69 [6.0]	23.25 [2.4]	0.08 ^c^
GWG ^a^	12.53 [4.3]	14.33 [5.3]	0.59 ^c^
GA ^a^	39.69 [1.2]	33.86 [1.4]	0.0002 ^c^
BW in g ^a^	3639.2 [492]	2150.4 [420]	<0.0001 ^c^
Sex (female)	8 (5)	6 (4)	1.0 ^d^
SGA ^b^	1/13	0/10	1.0 ^d^
Apgar score (1 min) ^a^	8.85 [0.38]	8.8 [1.0]	1.0 ^c^
Apgar score (10 min) ^a^	9.92 [0.28]	9.4 [0.52]	0.009 ^c^
Umbilical cord pH <7.2 ^b^	3/13	1/10	0.23 ^d^
C-section ^b^	5/13	3/10	1.0 ^d^
Complications during pregnancy ^b^	5/13	2/10	0.4 ^d^
Diabetes ^b^	1/13	0/10	1.0 ^d^
Gestational diabetes ^b^	4/13	2/10	0.66 ^d^

GWG gestational weight gain; GA gestational age; BW birth weight; SGA small for gestational age (BW < 10. percentile); ^a^ mean values with standard deviation, ^b^ cases/total number, ^c^ Mann–Whitney test, two-tailed, ^d^ Fishers exact test, two-tailed.

**Table 2:** Peptides derived from proteins other than ß- and κ-casein with higher intensities in preterm than in term samples were consistently quantified across both sample groups.

Peptide Sequence	Protein	Beta	Term (*n*)	Preterm (*n*)
KRGGYVWVETQATVIYNTKN	HIF 1-alpha	1.66	11	9
FPIMFPPNDPKAGTQGKCMPFFRAGFVCPTPPY	Lactoperoxidase	1.48	11	9
LLKQYFRDLPEPIFTSKLTTTFLQIYQLLP	StAR-related lipid transfer protein 8	1.34	10	10
KYNVEVKHIKIMTAEGLYRITEKKAFRGLTEL	Proto-oncogene vav	1.3	4	10

**Table 3:** Differentially abundant peptides (*n* = 35) in the breast milk of mothers with term and preterm infants with potential bioactive function according to Peptide ranker or MHPDB. Prediction was considered significant when the bioactive function score (*) was > 0.5. Additionally, peptides with an annotated function in MHPDB were added/labeled.

Sequence ID	Protein ID	Protein Name	Score > 0.5 *	beta	−log *p* Value
KLDLLMNKLVVVIFISVVLVCLVLAFGFGFS	O60423	Phospholipid- transporting ATPase	0.921	−1.41	5.61
PPPPPPPPHYPVLQRDLYM	Q9Y2G1	Myelin regulatory factor	0.920	−2.32	6.63
LPLPLLQPLM	P05814	Beta-casein	0.850	1.35	5.19
PPQPLWSVPQPKVL	P05814	Beta-casein	0.809	−1.41	5.07
PFFDPQIPKLTDLENLHLPL	P05814	Beta-casein	0.787	0.85	5.87
MPVLKSPTIPFF	P05814	Beta-casein	0.777	−1.64	4.98
FFDPQIPKLTDLENLHLPLP	P05814	Beta-casein	0.764	1.31	6.39
YPFVEPIPYGFLPQNIL	P05814	Beta-casein	0.759	1.39	6.04
IPFFDPQIPKLTDLENLHLP	P05814	Beta-casein	0.713	1.40	5.80
DPQIPKLTDLENLHLPL	P05814	Beta-casein	0.687	1.24	5.08
LQNPSESSEPIPLESREEYMNGMNR	P47710	α_S1_-casein	0.682	−1.28	5.35
SPTIPFFDPQIPK	P05814	Beta-casein	0.674	−1.23	5.25
QQGLGNLQPWMQ	Q13113	PDZK1-interacting protein 1	0.672	−1.92	5.00
SPTIPFFDPQIPKLTDL *^1^	P05814	Beta-casein	0.662	1.36	5.94
IYPFVEPIPYGFLPQN	P05814	Beta-casein	0.641	1.48	7.47
YPFVEPIPYGFLPQNI	P05814	Beta-casein	0.620	1.50	7.79
LWSVPQPKVLPIP	P05814	Beta-casein	0.615	−1.35	6.22
RLQNPSESSEPIPLESREEYMNGMN	P47710	α_S1_-casein	0.593	−1.52	7.14
LPLPLLQPLMQQVPQPIP	P05814	Beta-casein	0.585	1.65	9.36
PFFDPQIPKLTDLENLHLPLP	P05814	Beta-casein	0.585	1.20	4.97
LQNPSESSEPIPLESREEYMNGMN	P47710	α_S1_-casein	0.580	−1.44	6.57
HEDQQQGEDEHQDKIYPSFQPQP	P05814	Beta-casein	0.580	1.40	6.75
QQVPQPIPQTL	P05814	Beta-casein	0.564	−1.27	5.28
YPQIMQYVPFPP	P47710	α_S1_-casein	0.553	−1.51	5.26
DQQQGEDEHQDKIYPSFQPQP	P05814	Beta-casein	0.545	1.34	6.05
DPQIPKLTDLENLHLP	P05814	Beta-casein	0.544	1.36	8.61
LPLPLLQPLMQQVPQPIPQT	P05814	Beta-casein	0.540	1.60	8.08
YLAGRDLSRLPQLVGVSTPLQGGSNSAAAIGQSSGELR	P15291	Beta-1,4-galactosyl- transferase 1	0.539	−1.40	5.90
DLENLHLPLPLLQ	P05814	Beta-casein	0.535	1.50	8.04
VPYPQRAVPVQALLLN	P05814	Beta-casein	0.516	−1.41	5.78
IPKLTDLENLHLPLPLLQ	P05814	Beta-casein	0.512	1.43	6.35
PVLKSPTIPFFDPQIPKLT	P05814	Beta-casein	0.511	−1.70	8.03
HTVIFPLLNPIIYTLR	Q8NGD0	Olfactory receptor 4M1	0.508	−1.47	7.09
VMPVLKSPTIPFFDPQIPK	P05814	Beta-casein	0.505	−1.28	5.22
QELLLNPTHQIYPVTQPLAPVHNPISV *^2^	P05814	Beta-casein	0.123	−1.34	5.31

*^1^ stimulates proliferation, *^2^ antimicrobial.

## Data Availability

Restrictions apply to the availability of data generated or analyzed during this study to preserve mothers’ and infants’ confidentiality or because they were used under license.

## Supplementary Materials

The following supporting information can be downloaded at: https://www.mdpi.com/article/10.3390/nu17172724/s1, Table S1A: LC-MS/MS parameter; Table S1B: Proteome discoverer parameters for peptide/protein identification and intensity extraction; Table S2: Differential peptides; Table S3: Differential proteins.
