# Supplementary material for: Influence of Gestational Age on the Level of Functional Peptides (Peptidome) in Breast Milk
Source: Nutrients. 2025 Aug 22;17(17):2724. doi: 10.3390/nu17172724 (PMC12430670; doi:10.3390/nu17172724)
Supplement: Supplementary file 1 [file nutrients-17-02724-s001.zip › nutrients-3810082-supplementary/Suppl Table S1_LC-MS_parameters_modMGS.pdf]

Supplemental Table S1_A: LC-MS/MS parameter

reversed phase liquid chromatography (LC)	
reversed phase liquid chromatography	**Ultimate 3000 RSLC (Thermo Scientific)**
trap column	75 µm inner diameter, packed with 3 µm C18 particles (Acclaim PepMap100, Thermo Scientific)
analytical column	75 µm inner diameter, packed with 2.6 µm C18 particles (Accucore, 25 cm, Thermo Scientific)
flow rate	300 nl/min
column oven temperature	40°C
buffer system	binary buffer system consisting of 0.1% acetic acid in HPLC-grade water (buffer A) and 100% ACN in 0.1% acetic acid (buffer B)
gradient	gradient of buffer B: 2min 2% to 7 %, 32min 7% to 40%, 5min 40% to 60%, 1min 60 to 90%, 7 min 90%, 1 min 90% to 2%, 5 min 2%
Mass spectrometry	
instrument	Exploris 480 mass spectrometer (Thermo Scientific)
operation mode	data-dependent
electrospray	Nanospray Flex Ion Source
Full MS	
MS scan resolution	120,000
normalized AGC target	300 %=3E6
maximum ion injection time mode	auto
scan range	350 to 1650 *m/z*
spectra data type	profile
filter	
filter MIPS Mode	Peptide
filter minimum Intensity Threshold	5000

filter charge state	include charge state(s) 2-6
dd-MS2	
resolution	15,000
MS/MS AGC target	3e6
maximum ion injection time for the MS/MS scans	22 ms
number of MS/MS scans	Top 20
spectra data type	centroid
isolation window	1.4 Da
fixed first mass	110 m/z
dissociation mode	higher energy collisional dissociation (HCD)
normalized collision energy	Fixed, 30 %
dynamic exclusion	10 sec

Supplemental Table S1_B: Proteome discoverer parameters for peptide/protein identification and intensity extraction

Data analysis	
software	Proteome Discoverer 2.4
Analysis Type	Data dependent (DDA)
Serach engine used	Sequest HT
Min Peptide Length	6
Max Peptide Length	52
Missed Cleavages	Not applicable
Digest Typ	Specific
Enzymes / Cleavage Rules	Trypsin/P
Max Variable Modifications	5
Database Original File	Human milk DB (custom)
Fixed Modifications	Carbamidomethyl (C)
Variable Modifications	Acetyl (Protein N-term), Oxidation (M), conversion of N-terminal glutamate to pyroglutamate

Mass tolerance for precursor ions	10 ppm
Mass tolerance for fragment ions	0.02 Da
Threshold score peptides	FDR 0.05
Threshold score proteins	FDR 0.05
Threshold score for accepting proteinidentification	not applicable
Software/method used to evaluate site assignment	no PTM reported
